# Assessing and illuminating the Impact of Ultra‐Processed Food Intake on Alzheimer’s Disease‐A Systematic Review and Meta‐Analysis:

**DOI:** 10.1002/alz.095195

**Published:** 2025-01-09

**Authors:** Mostafa A. Khalifa, Jana Ahmed Elsead

**Affiliations:** ^1^ Faculty of Medicine, Cairo University, Cairo, Giza Egypt; ^2^ Faculty of Medicine, Cairo University, Cairo, giza Egypt

## Abstract

**Background:**

As there is currently no therapy available for Alzheimer’s disease, efforts are now focused on improving quality of life through various means, one of them is dietary intake. While several studies have demonstrated that consuming ultra‐processed food may contribute to the development of chronic diseases such as diabetes, there is insufficient evidence to directly link the consumption of ultra‐processed food to an increased risk of developing Alzheimer’s disease. However, it has been established that ultra‐processed foods have negative effects on biological mechanisms, such as oxidative stress and inflammation. Therefore, it is our role to clarify the impact of ultra‐processed food on Alzheimer’s disease

**Method:**

Our systematic review will include all records in Medline and Web of Science up to 2024 that describe the relationship between ultra‐processed food and Alzheimer’s disease as determined by the NOVA classification system. There were no limitations placed on the earliest search date. Studies not classified by NOVA were excluded, and we adhered to the PRISMA guidelines. Our systematic review is currently in progress and is expected to be completed by the end of 2024.

**Result:**

Our research yielded a total of 7560 observational studies. After removing 700 duplicate records, we screened the titles and abstracts of 6860 papers, excluding 6760. We then proceeded to review the full text of 100 papers, 10 of them met the inclusion criteria. A pilot study was conducted on 5 of these papers, revealing that all studies demonstrated a significant increase in the risk of developing Alzheimer’s disease among individuals with a high intake of ultra‐processed food.

**Conclusion:**

Based on our preliminary findings, we suggest that a high intake of ultra‐processed food may contribute to the development of Alzheimer’s disease. Further clinical trials are needed to illustrate the association between ultra‐processed food and Alzheimer’s disease.

